# Evaluation of ChatGPT Dermatology Responses to Common Patient Queries

**DOI:** 10.2196/49280

**Published:** 2023-11-17

**Authors:** Alana L Ferreira, Brian Chu, Jane M Grant-Kels, Temitayo Ogunleye, Jules B Lipoff

**Affiliations:** 1 Department of Dermatology Perelman School of Medicine University of Pennsylvania Philadelphia, PA United States; 2 Department of Dermatology University of Connecticut Health Center Farmington, CT United States; 3 Department of Dermatology University of Florida Gainesville, FL United States; 4 Department of Dermatology Lewis Katz School of Medicine Temple University Philadelphia, PA United States

**Keywords:** ChatGPT, dermatology, dermatologist, artificial intelligence, AI, medical advice, GPT-4, patient queries, information resource, response evaluation, skin condition, skin, tool, AI tool

## Introduction

Patients often turn to online resources for medical advice [1]. The chat-based artificial intelligence (AI) service ChatGPT has gained over 100 million users given its impressive responses to complex queries, and it is likely that patients are using it regularly [2]. For example, ChatGPT has demonstrated that it can provide largely appropriate medical advice to questions about cardiac disease [3].

Recently, ChatGPT has been upgraded to use the GPT-4 engine released in March 2023 and is more advanced than its predecessor, GPT-3.5 [4]. We aimed to assess the appropriateness of responses generated by ChatGPT using GPT-4 to common questions by dermatology patients.

## Methods

To assess the appropriateness of ChatGPT’s responses, 3 experienced dermatologists (JMG-K, TO, and JBL) designed questions to interrogate ChatGPT based on the prevalence of 6 common skin conditions [5] and common queries, supported by a literature review and the dermatologists’ professional perspectives. The questions were categorized into 7 groups: acne, atopic dermatitis, alopecia, psoriasis, rosacea, skin cancer, and miscellaneous. ChatGPT Plus was used to access GPT-4. This structured approach aimed to include a set number of 3 questions for each of the 6 primary skin conditions plus a miscellaneous category, resulting in 31 total questions when miscellaneous questions were added.

In April 2023, we queried ChatGPT with each question 3 times, yielding 93 responses. A new chat was initiated for each question to avoid prior context bias. The same 3 dermatologists independently assessed the responses, grading them as “appropriate” or “inappropriate” based on their expertise. Responses were then evaluated as “appropriate” or “inappropriate” based on majority agreement among the 3 dermatologists. In selecting 3 reviewers and repetitions for each question, we aimed to balance the need for a diverse range of evaluations with the practical considerations of managing the data.

## Results

ChatGPT generated 88% (82/93) appropriate and 12% (11/93) inappropriate responses (Table 1). Of the 31 questions, 16.1% (n=5) had an overall inappropriate response average, with at least 2 out of 3 dermatologists rating 2 out of 3 responses as inappropriate (Table 2). The appropriateness of ChatGPT’s responses was assessed based on the dermatologists’ perception of response accuracy and general public understanding. For instance, a question about common forms of alopecia prompted a reply that incorrectly stated alopecia areata was the most common cause; the response also did not mention traction and hair treatments as possible etiologies. All 3 dermatologists deemed this answer inappropriate due to its incompleteness and potential for misinformation.

**Table 1:** Proportion of appropriate and inappropriate ChatGPT responses by category.

Category	Questions, n	Total responses, n	Appropriate responses, n (%)	Inappropriate responses, n (%)
Acne	3	9	9 (100)	0 (0)
Atopic dermatitis	4	12	9 (75)	12 (25)
Alopecia	3	9	7 (77)	2 (22)
Psoriasis	4	12	10 (83)	2 (17)
Rosacea	3	9	9 (100)	0 (0)
Skin cancer	2	6	9 (100)	0 (0)
Miscellaneous	12	36	29 (80)	7 (20)
Total	31	93	82 (88)	11 (12)

**Table 2:** Example questions and ChatGPT responses.

Question	Response	Majority rating
What causes acne?	*Acne is a common skin condition that occurs when hair follicles become clogged with oil (sebum) and dead skin cells. Several factors contribute to the development of acne, including hormones, excess sebum production, dead skin cells, bacteria, inflammation, diet, and stress. It’s important to note that acne severity and triggers can vary greatly from person to person, and a combination of factors often contributes to the development of the condition.*	Appropriate
Will my child outgrow eczema?	*I am not a doctor, but I can provide you with some general information about eczema. Many children with eczema tend to see improvement as they grow older. In some cases, children may outgrow eczema completely by their teenage years or early adulthood. However, this is not true for everyone. Some individuals may continue to experience eczema symptoms into adulthood or have recurrent flare-ups throughout their lives. It is essential to work closely with your child’s pediatrician or dermatologist to manage their eczema effectively and monitor their progress over time.*	Appropriate
What are the best treatments for alopecia?	*I am not a doctor, but I can provide you with some general information on treatments for alopecia. Alopecia is a term that refers to hair loss, and it can have various causes, including genetic predisposition, autoimmune disorders, hormonal imbalances, or even stress. The most common type is alopecia areata, an autoimmune disorder that causes patchy hair loss. Some possible treatments for alopecia include topical treatments, oral medications, injections, light therapy, lifestyle changes, and alternative treatments. It’s important to note that the effectiveness of these treatments can vary depending on the individual and the underlying cause of hair loss. A healthcare professional can help determine the best course of treatment based on the specific case.*	Inappropriate

## Discussion

GPT-4–based ChatGPT shows potential as a public dermatology resource, producing 88% (82/93) appropriate responses. However, the responses were sometimes inaccurate, incomplete, or not evidence based, indicating the need for model improvements and emphasizing the importance of professional dermatologist consultations. For instance, ChatGPT incorrectly stated alopecia areata as the most common form of alopecia. This incorrect assertion may have been due to widely circulated information on the internet about alopecia areata compared to androgenetic alopecia despite the former being less common.

ChatGPT provided suitable answers for questions on acne, rosacea, and skin cancer but was inconsistent regarding diet, naturopathic remedies for eczema and psoriasis, antiaging treatments, skin care routines, and wound healing. These limitations may reflect ongoing debates and limited evidence in the dermatology community. This may also indicate a default bias toward producing answers rather than acknowledging no good answer exists, a flaw that could potentially perpetuate health misinformation. Indeed, ChatGPT’s performance on controversial or complex topics was suboptimal, often neglecting the lack of consensus or evidence.

Interestingly, ChatGPT provided different answers to the same question with varying completeness and accuracy. For instance, when asked about natural eczema treatments, one response suggested numerous unsupported methods whereas another advised consulting a health care professional.

Our results suggest that ChatGPT’s algorithmic curation does provide mostly relevant and accurate information in response to dermatologic queries. However, such tools may provide biased or inaccurate information. As such, we recommend that ChatGPT should not replace professional medical advice and should remain a supplementary informational tool for now. As AI advances, dermatologists must engage in developing clinical and patient-facing AI tools, considering public health and patient safety implications.

In the development of AI-based medical resources, it is crucial to rely on objective data, and we advocate for algorithms that are informed by rigorous, evidence-based sources such as PubMed, Web of Science, and Embase, weighted based on the standard assessments for the quality of research findings. Additionally, dermatologists should anticipate patients using ChatGPT for their skin-related questions and be familiar with the types of responses it generates.

## Abbreviations

AI: artificial intelligence
